# Effects of osteopathic manipulative treatment on maternal-fetal hemodynamics in third trimester pregnant women: A prospective study

**DOI:** 10.1371/journal.pone.0300514

**Published:** 2024-03-20

**Authors:** Maria Luisa Arruda Correia, Fernando Maia Peixoto Filho, Saint Clair Gomes Júnior, Guilherme Ramires de Jesus

**Affiliations:** 1 IFF/Fiocruz, Rio de Janeio, Brazil; 2 Department of Fetal Medicine, IFF/Fiocruz, Rio de Janeio, Brazil; 3 Department of Clinical Research, IFF/Fiocruz, Rio de Janeio, Brazil; 4 Department of Obstetrics, IFF/Fiocruz, Rio de Janeio, Brazil; Aga Khan University - Kenya, KENYA

## Abstract

**Objective:**

To evaluate the maternal-fetal hemodynamic effects after osteopathic manipulative treatment by measuring vital signs and Doppler velocimetry in third-trimester pregnant women.

**Materials and methods:**

This is a prospective study with pregnant women undergoing outpatient follow-up and hospitalized in a ward at Instituto Fernandes Figueira/Fiocruz, between August 2021 to August 2022, during the SARS-CoV-2 pandemic. This study was registered in REBEC under Register Number RBR-9q7kvg and approved by the ethics committee under number 32216620.0.0000.5269. The study population was composed of 51 pregnant women between 28 and 40 weeks of gestation, over 18 years of age, allocated in a single group. Pregnancies with multiple fetuses, malformations, premature rupture of the membrane, and active labor were excluded. The procedures evaluated maternal-fetal hemodynamics using three consecutive measures of ultrasound examination with Doppler velocimetry, and three maternal vital signs measured by an electronic blood pressure monitor.

**Results:**

Most vital signs changed after osteopathic treatment. However, only the systolic blood pressure (109.92 ±14.42 to 110.71±12.8, p = 0.033), diastolic blood pressure (79.8±11.54 to 77.57±9.44, p = 0.018) and heart rate (87.59±11.93 to 81.12±10.26, p = 0.000) in the sitting position, systolic blood pressure (110.75±13.26 to 108.59±13.07; p = 0.034) in the supine, and heart rate (83.22±11.29 to 80.39±11.0; p = 0.013) in left lateral decubitus reached statistical significance. The oximetry measures (98.55±0.64 to 98.67±0.68; p = 0.098) stayed stable during all three positions.

All artery values remained stable after treatment, and no statistically significant difference was recorded in the artery results.

**Conclusion:**

Responses to osteopathic treatment in women in the third trimester of pregnancy did not affect uteroplacental and fetoplacental circulation. However, some maternal vital signs had statistically significant results, with a decrease in diastolic blood pressure and heart rate, and an increase in systolic blood pressure in the sitting position, a decrease of heart rate in the left lateral decubitus position, and systolic blood pressure in the supine position. All the results observed were maintained in the normal parameters. The study responses attest to the safety of using the osteopathic manipulative treatment for the fetus and for pregnant women with comorbidities.

## Introduction

According to WHO recommendations in its antenatal care guidelines [1], women’s health care during the prenatal period requires the adoption of various therapeutic and diagnostic practices. Within the different types of care, ultrasound (US) is an important diagnostic and prognostic tool for maternal and fetal risks [2,3]. Ultrasound “before 24 weeks of gestation is recommended for pregnant women to estimate gestational age, improve detection of fetal anomalies and multiple pregnancies, reduce induction of labour for post-term pregnancy, and improve a woman’s pregnancy experience” [1]. Although the interpretation of the evidence that underpins the Guidelines may be influenced by individual circumstances, local protocol, and available resources [4], the US is an accurate form of image diagnosis to detect maternal and fetal risks.

In recent years, the US has become the most used technique to predict human development in the first trimester [5] in addition to becoming a fundamental tool in the diagnosis and treatment of congenital infections [4]. Its use, combined with doppler velocimetry (DV), allowed to understand the change in circulation during pregnancy and to improve the knowledge of the pathophysiological mechanisms associated [6] with fetal-placental diseases like stillbirth [7], fetal grown restriction, and premature birth [8].

The evaluation of the uterine (UtA), umbilical (UA), and middle cerebral (MCA) arteries allowed the detection of the risk of hemodynamic abnormalities in pregnancy [9], provided information on the perfusion of the fetoplacental circulation and specific fetal organs [10], in addition to allowing the prevention of perinatal complications in near-term fetuses [10,11].

The study of the flow velocity wave allows analysis of the entire cardiac cycle, being possible to distinguish the systolic and diastolic components. In pregnancies with normal evolution, the trophoblastic invasion, and the appearance of low-resistance vessels in the placental bed cause a decrease in the resistance to the umbilical artery blood flow [6].

The MCA is the vessel of choice when it is necessary to assess fetal cerebral circulation. Compared to other brain vessels, the MCA is easily identified, showing good reproducibility [12]. As normal pregnancy progresses, the MCA has high resistance to blood flow, reflecting the reduced diastolic flow velocity on Doppler [6]. In cases of placental insufficiency, MCA Doppler is used to investigate the existence of the central flow redistribution process. This pathophysiological process occurs whenever the fetus is in a hypoxemic environment. The conceptus, in these cases, promotes vasodilation of the cerebral vessels. This can be evaluated through the DV of that vessel, where it is possible to observe an increase in end-diastolic velocity, and consequently, a reduction in pulsatility index (PI) [13], or by the relationship between the PI of the MCA and UA [12].

Pregnancy is also subject to maternal hemodynamic problems, such as gestational hypertensive disorders and aorta-caval syndrome. The correlation between hemodynamic issues between mother and fetus can be expressed and detected through US examinations, as in the case of pre-eclampsia (PE) with changes in the fetal UA and MCA [14].

The search for non-invasive and non-drug practices that can help with maternal-fetal hemodynamics made manipulative treatments a choice. Osteopathic manipulative treatment (OMT) identified by TART parameters (Tenderness, Asymmetry, Range of motion, Tissue texture abnormalities) [15], is a non-invasive therapy, centered on the individual and body self-regulation, where practitioners diagnose and treat somatic dysfunctions through their hands [16].

Several manipulative treatments are used in pregnant women [17–22] due to pain conditions that routinely occur among them. In recent years, the demand for integrative and complementary practices in the prevention and treatment of various diseases has led the World Health Organization (WHO) to recommend that member States formulate laws to integrate these practices into their health systems [23].

Some research has already observed neurophysiological effects of OMT on blood pressure (BP) [24], on increased brachial blood flow in patients with heart failure [25], and changes in cardiovascular autonomic parameters in athletes [26]. Although there is a study on hemodynamic control in pregnant women after OMT [27], research aimed at ensuring the safety of OMT on maternal-fetal hemodynamics has not yet been carried out. This study aimed to evaluate the maternal-fetal hemodynamic effects after OMT by measuring vital signs and US examinations with DV in pregnant women in the third trimester.

## Materials and methods

### Study design

This is a prospective study aimed to assess the variability of maternal-fetal hemodynamics after OMT. Fifty-two women in the third trimester of pregnancy were screened and allocated in a single group with a single treatment. After signing an informed consent statement attesting to their awareness and willingness to enter the study, they authorized access to information on clinical, obstetric, and demographic data contained in medical records. The procedures performed evaluated maternal-fetal hemodynamics with the use of US and DV, and maternal vital signs were measured by an electronic BP monitor.

### Study location, participants, and inclusion and exclusion criteria

The study was carried out between August 2021 and 2022, during the SARS-CoV-2 pandemic, with a convenience sample formed by patients from the pregnant women’s ward and the prenatal clinic of Instituto Fernandes Figueira (IFF-Fiocruz), in the city of Rio de Janeiro, RJ. The research was carried out at the fetal medicine department of the same Institute, and the IFF Research Ethics Committee approved the project under number 32216620.0.0000.5269 and was registered in REBEC (Brazilian Registry of Clinical Trials) under Register Number RBR-9q7kvg.

### Population

Inclusion criteria were pregnant women between 28 and 40 weeks of gestation, over 18 years of age, with prenatal outpatient follow-up or hospitalized in the pregnant women’s ward at IFF/Fiocruz from July 2021 to June 2022. Exclusion criteria were pregnant women under 18 years of age, twins, malformation, premature rupture of the membrane, and active labor.

All clinical, obstetric, and demographic data of the pregnant women were taken from the institution’s medical records, which included laboratory tests, and diagnoses given by obstetricians, psychiatrists, and psychologists from the IFF.

### Informed consent statement

All patients included in the study had signed a written term of free and informed consent statement (S1 File).

### Evaluated variables

Primary measures were categorized into independent and dependent variables. The independent variables measured were the mean BP, heart rate (HR), and oximetry. The dependent variables were the mean PI of the UA, MCA, and UtA. The cutoff points for predicting risk to the conceptus followed the parameters of The Fetal Medicine Foundation [28]. The secondary measures observed were demographic data (age, education, gestational age, race, marital status, and family income).

### Gestational age (GA)

The GA was determined by the date of the first-trimester ultrasound according to the recommendations of the International Federation of Gynecology and Obstetrics (FIGO, 2021) [29].

### Hypertensive disorders of pregnancy

The Hypertensive disorders of pregnancy were defined by the American College of Obstetricians and Gynecologists (ACOG, 2020) [30] criteria which include gestational hypertension (GH) and PE, with the presence of GH being diagnosed when systolic BP ≥ 140 mm Hg and/or diastolic pressure ≥ 90 mm Hg, at least twice in 4 hours, starting in the 20th week of pregnancy and in women with previously normal blood pressure. PE is a type of gestational hypertensive disorder that occurs most frequently after 20 weeks of gestation or near term, associated with signs and symptoms that may or may not be accompanied by proteinuria [30].

### BMI measurements

BMI referred to the data collection day and was calculated using three measurements: height, weight, and gestational age. According to the Institute of Medicine (IOM, 2009) [31] the cutoff points for obesity are: underweight (< 18.5 kg/m^2^), adequate weight (18.5–24.9 kg/m^2^), overweight (25 to 29.9 kg/m^2^), grade 1 obesity (30–34.9 kg/m^2^), grade 2 obesity (35 and 39.9 kg/m^2^) and grade 3 obesity (≥40 kg/m^2^).

### Outcomes

The primary outcome is the assessment of UA through US examination with DV. Secondary outcomes were maternal vital signs, measured through systolic and diastolic BP, heart rate and oximetry, and the assessment through US examination with DV of UtA and MCA.

### Procedures

The pregnant women who participated in the research followed the same procedure and received a single treatment. Initially, a US examination with DV was performed to assess fetal circulation. After the exam, BP, HR, and oximetry were measured. An OMT session was then held, after which maternal vital signs assessment was repeated and finally fetal circulation assessment.

### Ultrasound evaluation by DV

All exams were performed on Volusom E10 and E8 GE equipment. Both devices have the characteristics recommended by the ISUOG [32] for performing obstetric examinations. The examinations were performed by two non-blinded doctors certified in fetal medicine and with experience in clinical research.

Ultrasound evaluation with DV recorded three similar consecutive pulse waves to visualize the UtA, UA, and MCA and assess the flow impedance with the PI through the transabdominal approach. Insonation of all vessels in this study was obtained during the absence of fetal respiratory and body movements and following the recommendations of The International Society of Ultrasound in Obstetrics and Gynecology (ISUOG) [32].

### BP assessment

The BP of all patients was measured before and after OMT. The BP measurement followed the clinical trial guidelines, which guide the use of electronic monitors. The OMROM HEM 7320 device was used, was validated and reached the required standards, [33] and recognized by the Brazilian Society of Cardiology.

Blood pressure was measured before and after OMT in three positions, sitting, supine position, and left lateral decubitus, the first measurement being after a minimum rest period of five minutes. The patient’s measurement in the sitting position follows the manufacturer’s guidelines, which recommends the use of the cuff on the left arm, above the elbow, with the arm and feet resting on a surface. In dorsal decubitus, the arm was kept along the body, and in lateral decubitus it stretched forward.

All equipment used during the research (Volusom E10 and E8 GE and OMROM HEM 7320) are certified by ANVISA, the Brazilian National Health Surveillance Agency.

### OMT

All patients were always wearing a mask during the research and received the same protocol, prepared for the study, which included osteopathic techniques for balancing ligament tensions (BLT), myofascial, muscle, and cranial energy. High-velocity, low-amplitude (HVLA) techniques were excluded. The OMT sessions ranged from 30 to 40 minutes and were performed by a single D.O osteopath with 20 years of practice, non-blinded. The pregnant women were instructed to maintain standard obstetric and pharmacological treatment. The OMT protocol with the techniques used is described in the (S2 File).

### Objective and hypothesis

This study aims to evaluate the effects of OMT on the maternal-fetal hemodynamics of pregnant women in the third trimester treated in a tertiary fetal risk unit. It is hypothesized that OMT is safe.

### Sample calculation

The sample size was calculated considering the umbilical arterial pulsatility index. We estimated a minimum of 43 participants to observe a clinically significant difference of at least 0.20, with a standard deviation of 0.35, a confidence level of 95% and a power of 80%.

### Statistical analysis

Statistical analysis was carried out using SPSS version 23. The numeric variables were analyzed by mean and standard deviation and the categorical variables by their absolute and relative frequencies. The Wilcoxon test for paired samples was performed to evaluate significant differences in the parameters considered pre- and post-TMO. All analyses were performed considering a significance level of 0.05.

## Results

**Fig 1:**
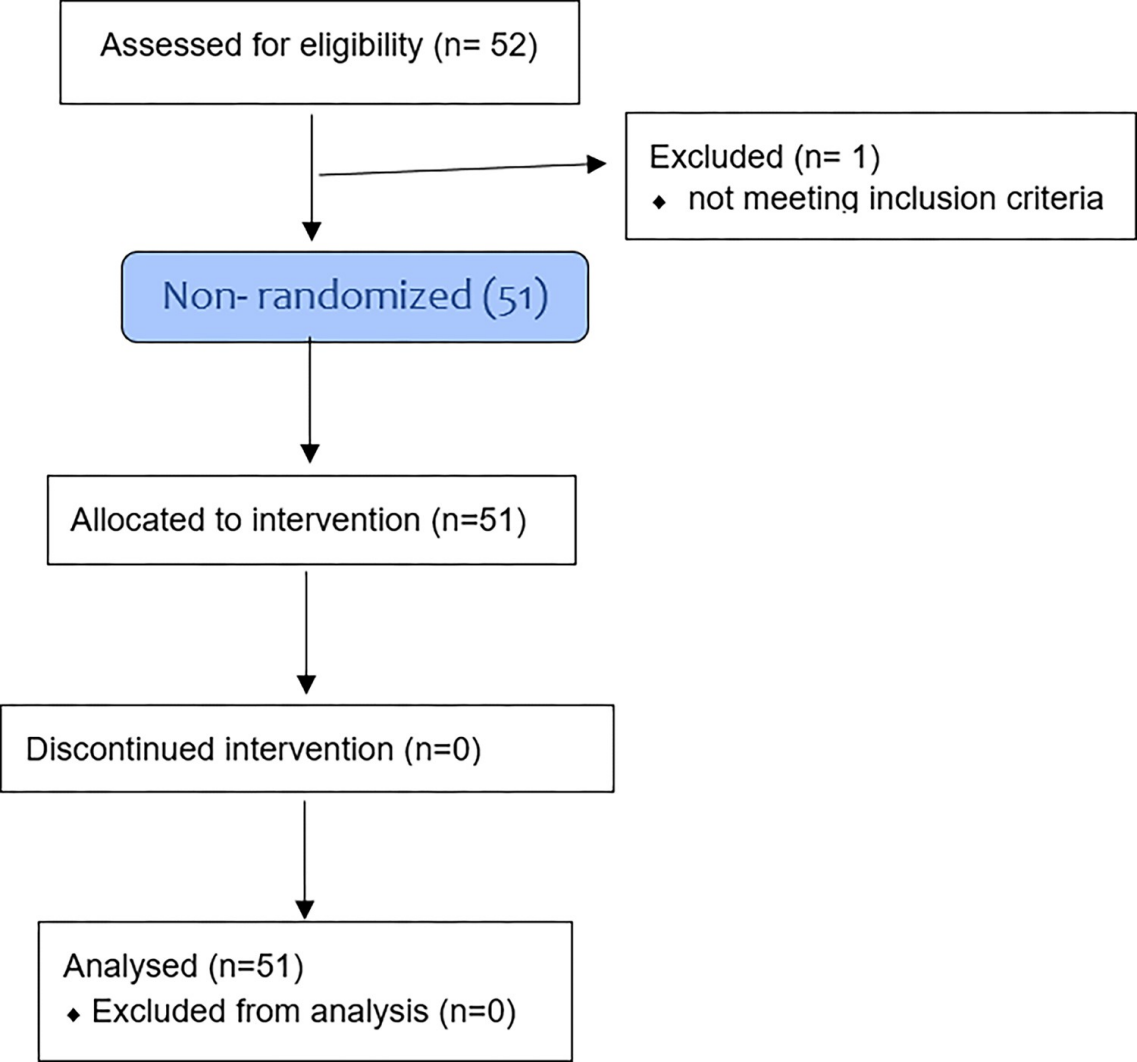
Flow diagram.

Fig 1 contains the flowchart with the number of patients included in the study. The study was carried out between August 2021 and 2022 and 52 women in the third trimester of pregnancy were screened to a single treatment. One pregnant woman was excluded due to an infectious disease (scabies). There remained 51 women who, before and after OMT, underwent procedures that evaluated maternal-fetal hemodynamics by means of an ultrasound examination with DV, whereas maternal vital signs were measured by an electronic BP monitor. Each subject served as their own control.

### Clinical and sociodemographic characteristics and hemodynamic risks of the sample

The women who made up the sample came from the prenatal clinic (38) and the pregnant women’s ward (13). The sample is composed of normotensive (34) and hypertensive (17) pregnant women (chronic or with gestational hypertensive disorders). The age range among pregnant women was 29.37 ± 6.6 and the mean gestational age (GA) was 34.67 ± 3.28. The majority of patients (60.8%) were obese, and the mean BMI was 31.05 ± 5.34. The sample is predominantly white (53%), and there were more single (53%) than married women (47%) (Table 1).

**Table 1:** Clinical and sociodemographic characteristics of the pregnant women in the sample.

**Variables**	**Total = 51** *
**Normotense**	**34 (66.7%)**
**GHD**	**17 (33.33%)**
**Hospitalized in the pregnancy ward**	** **
**Yes**	**13 (25.5%)**
**No**	**38 (74.5%)**
**Color/Race**	** **
**white**	**27 (53%)**
**black**	**8 (15.7%)**
**brown**	**16 (31%)**
**Marital status**	** **
**married**	**24 (47%)**
**single**	**27 (53%)**
**Age**	** **
**Age (mean and SD)**	**29.37 ± 6.66**
**≤19**	**2 (3.9%)**
**20–34**	**36 (71%)**
**35–39**	**11 (22%)**
**≥40**	**2 (3.9%)**
**BMI**	** **
**BMI (mean and SD)**	**31.05 ± 5.34**
**Under Weight (< 18 kg/m^2^)**	**0 (0%)**
**Suitable Weight (18.5–24.9 kg/m^2^)**	**3 (5.9%)**
**Overweight (25 to 29.9 kg/m^2^)**	**17 (33%)**
**grade 1 obesity (30 to 34.9 kg/m^2^)**	**18 (35%)**
**grade 2 obesity (35 and 39.9 kg/m^2^)**	**10 (20%)**
**grade 3 obesity (≥40 kg/m^2^)**	**3 (5.9%)**
**GA**	** **
**GA (mean and SD)**	**34.67 ±3.28**
**28–30**	**4 (12%)**
**31–33**	**7 (21%)**
**34–36**	**12 (35%)**
**37–38**	**6 (18%)**
**39–40**	**5 (15%)**

* values measured in mean ± SD and percent.

Abreviations: GHD = gestational hypertensive disorders; BMI = body mass index; GA = gestational age.

The patients admitted to the ward were mostly hypertensive (69%) and those with gestational diabetes mellitus (GDM) were almost exclusively normotensive (77.7%), with 30% being hospitalized due to increased blood glucose (Table 2).

**Table 2:** Frequency of hemodynamic risk factors in the sample.

Comorbidities	Total	Percent
**Alloimmunization^1^**	**6**	**11.8%**
BMI *	**31**	**60.8%**
**GHD^2^**	**17**	**33.3%**
**GDM^3^**	**11**	**21.6%**
**HIV**	**3**	**5.9%**
**Psychiatric condition/anxiety ⁴**	**8**	**15.7%**
**Neurological disease (epilepsy) ⁵**	**1**	**2%**
**Syphilis ⁶**	**1**	**2%**
**Toxoplasmosis ⁹**	**2**	**3.9%**

Abbreviations: BMI = body mass index; GHD = gestational hypertensive disorders; GDM = Gestational Diabetes mellitus.

Values according to IOM.

^1^ One was hospitalized.

^2^ The group includes chronic hypertensive, and GHD (PE and gestational hypertension). Eleven were hospitalized in the pregnancy ward, and 14 were obese (82.3%).

^3^ In this group 4 were hospitalized, three were hypertensive, and 9 were obese.

⁴ In this group 4 were hospitalized and all were hypertensive.

⁵ she presented the condition in childhood.

⁶ She was hospitalized and also obese, had GDM, PE, and alloimmunization.

⁹ One had GDM association.

Six cases of maternal-fetal alloimmunization were in the group, but none of the fetuses presented anemia. The only hospitalization was due to other causes. Among the 8 patients with anxiety, four were hospitalized. There was one case of epilepsy in outpatient follow-up and without seizures for years.

Infectious diseases such as syphilis, HIV, and toxoplasmosis affected 11.8% (6) of the sample. Those with HIV had undetectable rates, and the pregnant woman with syphilis was also obese and had GDM, PE, and alloimmunization.

### Results of hemodynamic factors before and after OMT

Table 3 shows the measurements taken before and after OMT, expressed as mean values with the respective standard deviations and p-value.

**Table 3:** Comparison of mean maternal-fetal hemodynamics measured before and after OMT.

Variables	Valid	Missing Data	Before OMT = 51*	After OMT = 51*	p-value
**SBP sitting**	51	0	109.92 ± 14.42	110.71 ± 12.8	0.033
**DBP siting**	51	0	79.8±11.54	77.57 ± 9.44	0.018
**HR sitting**	51	0	87.59±11.93	81.12 ± 10.26	0.000
**SBP supine**	51	0	110.75 ± 13.26	108.59 ± 13.07	0.034
**DBP supine**	51	0	77.47 ± 11.37	77.76 ± 9.94	0.588
**HR supine**	51	0	84.24 ± 11.12	82.39 ± 11.39	0.098
**SBP LLD**	51	0	108.80 ± 14.18	111.18 ± 14.84	0.146
**DBP LLD**	51	0	77.88 ± 11.93	78.45 ± 11.41	0.988
**HR LLD**	51	0	83.22 ± 11.29	80.39 ± 11	0.013
**oximeter^1^**	51	0	98.55 ± 0.64	98.67±0.68	0.098
**Umbilical Artery 1**	49	2	0.88 ± 0.2	0.84 ± 0.17	0.12
**Umbilical Artery 2**	48	3	0.9±0.17	0.86 ± 0.19	0.198
**Umbilical Artery 3**	48	3	0.89 ± 0.18	0.86 ± 0.15	0.207
**Middle Cerebral Artery 1**	50	1	1.9 ± 0.55	1.91 ± 0.76	0.557
**Middle Cerebral Artery 2**	48	3	1.96 ± 0.51	1.92 ± 0.52	0.874
**Middle Cerebral Artery 3**	47	4	1.91 ± 0.52	1.82 ± 0.45	0.167
**Right Uterine Artery 1**	49	2	0.78 ± 0.32	0.85 ± 0,4	0.056
**Right Uterine Artery 2**	48	3	0.82 ± 0.37	0.83 ± 0.38	0.363
**Right Uterine Artery 3**	48	3	0.76 ± 0.35	0.79 ± 0.37	0.339
**Left Uterine Artery 1**	48	3	0.83 ± 0.36	0.81 ± 0.35	0.968
**Left Uterine Artery 2**	48	3	0.82 ± 0.36	0.79 ± 0.37	0.708
**Left Uterine Artery 3**	46	5	0.8 ± 0.37	0.78 ± 0.32	0.953

* All data was measured in mean ± SD.

Abreviations: SBP = systolic blood pressure; DBP = diastolic blood pressure; HR = heart rate; LLD = left lateral decubitus.

^1^ The measures in all position was stable. That´s why only one measure was cited.

Three measurements were taken of each of the vital signs analyzed. Excluding oximetry, all the vital signs in a sitting position had statistical significance. The systolic blood pressure (SBP) rose after OMT from 109.92 ±14.42 to 110.71±12.8 (p = 0.033), and diastolic blood pressure (DBP) decreased from 79.8±11.54 to 77.57±9.44 (p = 0.018). However, the index with the most remarkable change was HR, which went from 87.59±11.93 to 81.12±10.26 (p = 0.000).

Although SBP (110.75±13.26 to 108.59±13.07; p = 0.034) and HR (84.24±11.12 to 82.39±11.39; p = 0.098) have decreased in the supine position, only SBP has statistical significance. The DBP (77.47±11.37 to 77.76±9.94; p = 0.588) stayed stable.

In the left lateral decubitus (LLD), SBP (108.80±14.18 to 111.18±14.84; p = 0.146) and DBP (77.88±11.93 to 78.45±11.41; p = 0.988) rose after OMT, but HR (83.22±11.29 to 80.39±11.0; p = 0.013) decreased and had statistical significance. The oximetry measures (98.55±0.64 to 98.67±0.68; p = 0.098) before and after OMT stayed stable during all three positions.

### Variability of arteries

Three consecutive measurements were taken in the supine position to assess fetal circulation. Although the differences observed in PI of arteries, the examinations performed did not achieve statistical significance. The mean PI of UA decreased after OMT. Almost the same process was observed with MCA. However, all measures of right UtA increase while those of left UTA decrease after OMT (Table 3).

### Adverse events

No adverse events occurred or were reported.

## Discussion

Sample analysis showed statistically significant results after OMT in all maternal vital signs in the sitting position, as well as SBP in the supine position and HR in the LLD position. The results of fetal circulation were not statistically significant, and all fetal-maternal measurements remained within normal parameters [34,35].

The data also made it possible to observe a heterogeneous population with varied clinical conditions, which could be hospitalized or under outpatient follow-up at the IFF, a high-risk tertiary unit specialized in women’s health. Collection took place during the SARS-CoV-2 pandemic in Brazil, a period marked by an impact on the mental health of pregnant women [36]. Many of the pregnant women had one or more comorbidities, with the potential to affect maternal-fetal hemodynamics and likely to be detected in an assessment by DV.

Regarding the particularities of the sample, the 17 pregnant women present in the study with hypertensive conditions had a condition associated with obesity (82.3%) and anxiety (47%). Arterial hypertension is a pathology that impacts the uteroplacental circulation, leads to intrauterine fetal growth restriction and directly affects the UA and MCA [14]. According to studies, the UA and MCA are essential vessels for predicting adverse perinatal outcomes, and the PI plays a role in assessing and monitoring fetal oxygenation, low neonatal hydrogen potential (PH), and admission to neonatal units [37]. Although the sample did not present severe acute hypertension or IUGR during the study period, it is essential to highlight that hypertension is associated with generalized arteriolar vasoconstriction and reduced uteroplacental flow and consequent oxygen supply deficit in areas of maternal-fetal exchange, subjecting the fetus to hypoxia [38].

Obesity wasn´t a particularity of GHD in the sample. It was present in most pregnant women (60.8%) and is another parameter with the power to interfere with the UA PI, interfering harmfully with the fetus-placental vessels [39]. Even in uncomplicated pregnancies, the bigger is the maternal BMI, the higher the resistance in UA [39]. Obesity is also related to increased intra-abdominal hypertension, which represents a risk of venous congestion and abdominal compartment syndrome [40].

The sample also consisted of 16% of patients with anxiety/psychiatric conditions. According to the literature, prenatal stress directly correlates with premature birth, PE, IUGR, and changes in maternal-fetal circulation. However, evidence for an association between prenatal stress and maternal and fetal circulation changes during pregnancy remains inconclusive [41].

Other pathologies in the population, such as alloimmunization, also compromise fetal circulation and are associated with increased blood flow velocity due to fetal compensations for hemodynamic adaptations [42]. Viruses, such as HIV virus and protozoan like toxoplasmosis, can be detected by changes in UA [43,44].

Cases of diabetes were associated with obesity (82%). GDM is a pathology that affects the fetal cardiovascular system, especially the umbilical vein and hepatic circulation, in addition to increasing the chances of developing PE and eclampsia [45]. Despite the mothers’ clinical condition, all fetuses in our sample had normal hemodynamics.

Concerning maternal hemodynamics, except for oximetry, the mean values of all vital signs in the three positions decreased after OMT. Considering the situation of the sample subject to pathologies with cardiovascular risks, the significant results in the sitting, supine, and LLD positions within normal values credit the safety of OMT in pregnant women with maternal-fetal risk. The reference indices used [30] showed that the mean values of systolic BP, as well as HR, are necessary signals for predicting maternal health. Small threshold changes often affect clinical scores identifying physiological deterioration [46]. During pregnancy, the increase in myocardial alpha receptors leads to an increase in heart rate [46], which makes pregnant women more apt to responses such as the increase in plasma and cardiac output induced by the pregnancy state, and on the other hand, more responsive to situations of stress.

It is important to highlight that despite pregnant women being more subject to changes in HR, the stress experienced during the pandemic period, the situation of part of the sample admitted to the pregnancy ward, and the number of patients with anxiety/psychiatric conditions (16%), the best results found occurred at this marker.

Pregnant women in the third trimester also are subject to compression of the aorta/caval system, which makes blood return and cardiac output difficult [47]. In addition, uterine development would alter IAP, which would generate patterns of abdominal hypertension (AIH), accommodated by a significant portion of pregnant women. Adaptation to increased IAP would be correlated with the ability to adapt abdominal compliance to increased pregnancy content [40,48–52].

The osteopathic techniques used in the protocol aimed to balance myofascial tensions, mainly correlated with the abdominal compartment, composed of bones and muscles, in which the thoracic and pelvic diaphragms play an essential role. The thoracic diaphragm is a muscle correlated with venous return and maintenance of thoracic and abdominal pressures [39]. In pregnant women, the cephalad retraction of the diaphragm interferes with its functionality, decreasing the respiratory amplitude and affecting the venous flow.

Touch manual therapies aim to untangle fascial restrictions, freeing body structures for comfortable positioning [53]. Through touch and movement, the osteopath can access not only the myofascial, joint, and lymphatic components but also affect the dynamics of blood fluids. OMT can directly influence the dynamics of blood flow through its action on related myofascial components such as blood vessels [54].

The hemodynamic responses produced by touch are also associated with the autonomic nervous system (ANS). Effects such as vasodilation, smooth muscle relaxation, increased circulation, and tissue changes are due to the interaction of touch with the ANS [26].

Neurophysiological responses that OMT is capable of producing on the diaphragm can influence the indirect hemodynamic responses resulting from the relationship between the diaphragm muscle and the vascular network. Increased intrathoracic pressure, which has one of its central regulators in the diaphragm, can activate the baroreceptor reflex system, leading changes in the arterial circulation [55]. Therefore, osteopathy is a possible way of controlling external structural agents that can lead to dysfunctions in the diaphragms and compressions of vascular-nervous structures [15].

The reflex actions of OMT on the ANS, as well as on the myofascial, arterial, and venous vascular network, could explain the changes statistically significant observed in the vital signs of pregnant women.

The OMT responses in the fetal circulation were observed through US with DV, and the pulsatility index (PI), which is the ratio of the result of the difference between the maximum systolic velocity and minimum diastolic velocity divided by the average velocity [12] was the index chosen for the data analysis. The PI is the most used index in clinical practice because it is more consistent from a mathematical point of view. It shows a linear correlation with vascular resistance as opposed to RI, which shows a parabolic relationship with increased vascular resistance. The PI value is not compromised when absent or inverted diastolic values are obtained [32].

UA was the vessel chosen as a clinical safety parameter of the research because the UA study provides information on the perfusion of the fetoplacental circulation and specific fetal organs [10]. Abnormal UA Doppler results, characterized by increased PI, arise when at least 30% of the villous tree is obstructed. Progression to absent (zero diastoles) or reversed (reverse diastole) flow occurs when 70% of the placental area is affected [56]. These findings reflect severe placental failure, in which almost all the vessels of the fetal-placental circulation are obstructed and are associated with a high incidence of fetal acidemia and neonatal morbidity [13].

The investigation of the MCA and its relationship with the UA, brain-placental relationship, did not show any change in resistance before and after OMT. In the patients studied in the present study, there was no significant change in the umbilical artery PI before and after OMT, ensuring the safety of its use in this fetal compartment.

The abnormal placentation that characterizes preeclampsia is associated with increased resistance in the uteroplacental circulation. Based on this assumption, the analysis of UtA’s DV in the assessment of preeclampsia risk has been extensively studied, initially in the second trimester and later at the beginning of pregnancy. Quantitative assessment demonstrates the increase in PI in cases with increased risk of placental insufficiency, intrauterine growth restriction, and PE [57]. As in the other territories, there was no significant change in velocity indices in UtA before and after OMT.

However, it is essential to highlight that the values of all pregnant women who participated in the research were within normal limits before and remained so after using OMT. Therefore, the non-change in data values observed after OMT is a favorable aspect of its use in pregnant women with comorbidities.

### Limitations and strengths

One of the limitations of the study lies in the heterogeneity and size of the sample. It would be interesting if a comparative study could be carried out between normotensive pregnant women and those with GHD. The effects on maternal-fetal hemodynamics would be better assessed in different clinical conditions. Finally, a more robust sample would be able to better assess the strength of the evidence found.

The strength of the present study lies in the originality of the research. To date, no publications have included pregnant women with diverse clinical conditions and associated comorbidities. There are also no studies on the effects of OMT on uteroplacental and fetoplacental circulation. Studies that evaluated OMT and hemodynamics in pregnant women [27] were restricted to the effects on maternal hemodynamics. Proving the safety of using osteopathic techniques paves the way for new research with OMT and pregnant women with adverse clinical conditions.

## Conclusion

Based on the research data, statistically significant changes were found in the vital signs of women in the third trimester of pregnancy in the sitting position, in SBP in the supine position, and HR in the LLD after OMT. However, data on uteroplacental and fetoplacental circulation were not statistically significant.

The responses of all observed data remained within the usual standards after OMT, attesting to the safety of its use for fetuses and pregnant women with comorbidities. However, the significant results of pregnant women’s vital signs open space for the investigation of new studies on the effects of OMT on maternal hemodynamics.

## Supporting information

S1 Checklist TREND statement checklist. (DOCX)

S1 Data (XLSX)

S1 File (DOCX)

S2 File Osteopathic techniques. (DOCX)
