# Supplementary material for: Effects of osteopathic manipulative treatment on maternal-fetal hemodynamics in third trimester pregnant women: A prospective study
Source: PLoS One. 2024 Mar 20;19(3):e0300514. doi: 10.1371/journal.pone.0300514 (PMC10954147; doi:10.1371/journal.pone.0300514)
Supplement: S1 File — (DOCX) [file pone.0300514.s003.docx]

MINISTRY OF HEALTH - National Health Council - National Research Ethics Committee - CONEP

RESEARCH PROJECTS INVOLVING HUMAN BEINGS

**Research project:**

Effects of osteopathic manipulative treatment on the hemodynamics of pregnant women

**Preliminary Information**

**Lead Researcher**

CPF/Documento: 041.303.147-00

Phone number: 2122080364

Name: Fernando Maia Peixoto Filho

E-mail: [peixotofilho@iff.fiocruz.br](mailto:peixotofilho@iff.fiocruz.br)

**Proponent Institution**

CNPJ: 33.781.055/0002-16 Institution Name: Instituto Fernandes Figueira - IFF/ FIOCRUZ - RJ/ MS

**Is it an international study? No**

**Assistants**

CPF/Document 847.074.887-49

Name

MARIA LUISA ARRUDA CORREIA

**Research Team**

CPF/Documento Nome

013.852.057-76 Saint Clair Gomes Junior

847.074.887-49 MARIA LUISA ARRUDA CORREIA

**Study area**

**Significant Areas of Knowledge (CNPq)**

**Major Area 4. Health Sciences**

**Main Purpose of the Study (WHO)**

**Clinical**

**Public Research Title:** Effects of osteopathic manipulative treatment on the hemodynamics of pregnant women

Public Contact

Name Phone

847.074.887-49 MARIA LUISA ARRUDA CORREIA 21988648158 maluarruda842@gmail.com

Scientific contact: Fernando Maia Peixoto Filho

**Study Design / Financial Support**

Study Design: Intervention/Experimental

**Health conditions or problems**

gestational hypertension

hypertensive pregnant woman

**General Descriptors for Health Conditions**

**ICD1-10: International Classification of Diseases**

O13 Gestational [pregnancy-induced] hypertension without significant proteinuria

**DeCS: Health Science Descriptors**

hipertensão gestacional hypertension, pregnancy induced hipertensiòn inducida en el embarazo hipertensão induzida pela gravidez

**Specific Descriptors for Health Conditions**

**ICD1-10: International Classification of Diseases**

O13 Gestational [pregnancy-induced] hypertension without significant proteinuria

**DeCS:Health Science Descriptors**

hipertensão gestacional hypertension, pregnancy-induced hipertensión inducida en embarazo

**Nature of Intervention**

Another integrative and complementary practice, manipulative treatment

**Intervention Descriptors**

osteopathic manipulation

**ICD list**

M90.8 Osteopathy in other diseases classified elsewhere

**DECS Description DECS**

**list of DECS**

osteopathic manipulation, osteopathic manipulation, osteopathic

**Drawing:**

cohort study

**Financial support**

Self-funded

**Key words**

integrative and complementary practices

fetal risk

osteopathy

osteopathic manipulation

**Study Details**

**Summary:**

The study seeks to evaluate the effects of osteopathic manipulative treatment (OMT) on the diaphragms in the hemodynamics of chronic and gestational hypertension in hypertensive pregnant women. OMT is already widely implemented as a therapy in normotensive pregnant women, including several RCTs proving its effectiveness in controlling musculoskeletal conditions. The search for OMT throughout pregnancy is a reality in countries like the USA and Australia. Australian observational studies (1,2) with pregnant women attest to the many women who seek and good results. In the USA, there was a reduction in complications during pregnancy and childbirth in a multicentric survey in osteopathic hospitals on pregnant women who had their prenatal care with OMT monitoring (3). Already in an ECR with a therapy-sacral skull, a specialty of osteopathy, a slight improvement in the pelvic pain of the pregnant women was observed (4). all studies do not report intercurrences or risks for pregnant women submitted to BMT throughout pregnancy. The present study, however, seeks to evaluate the possibility of improving perfusion with BMT in hypertensive pregnant women. As osteopathy is an integrative practice that does not use medication, positive research results could place it as an auxiliary therapy in controlling hypertension in hypertensive pregnant women.

The study provides a follow-up of the pregnant women who adhere to the research, from their acceptance until the end of the pregnancy. Therapy will be performed on the same days as the routine IFF prenatal consultations

References Requested

1- FRAWLEY, J. et al. Prevalence, and characteristics ofwomen who consult with osteopathic practitioners during pregnancy; a report from the Australian Longitudinal Study on Women’s Health (ALSWH).

Journal of Bodywork and Movement Therapies, v. 20, no. 1, p. 168–172, Jan. 2016.

2- SMITH, S. A study into osteopathic treatment of pregnant women in NSW and Queensland. PhD Thesis—[s.l.] Victoria University, 2005.

3-KING, H. H. et al. Osteopathic Manipulative Treatment in Prenatal Care: ARetrospective Case Control Design Study. P. 6, [n.d.].

4- ELDEN, H. et al. Effects of craniosacral therapy as adjunct to standard treatment for pelvic girl pain in pregnant women: a multicenter, single blind, randomized controlled trial. Acta obstetricia et gynecologica Scandinavica, v. 92, no. 7, p.775–782, 2013.

**Introduction**

Gestational hypertensive disorders (GHD) are a worldwide public health problem. They affect 6 to 8% of women and are the most common cause of pregnancy-related morbidity and mortality worldwide (1,2). GHD represents the main reason for fetal obituary and neonates with perinatal hypoxia (3,4). In Brazil, according to data from the 7th Brazilian guideline on arterial hypertension, the disease affects 7.5% of pregnancies and about 2% progress to eclampsia (5). The diagnosis of the hypertensive disease is given by the presence of high blood pressure. Systolic (SBP) at 140 mmHg and diastolic (DBP) at 90 mmHg, found in an average of at least two measurements in a period of 15 min (1–3). Gestational hypertensive disorders (GHD) are classified as pre-existing when the pregnant woman already presents a condition hypertensive before pregnancy; gestational hypertension (HG), when it appears during pregnancy and pre-eclampsia (PE). AS DHG usually is associated with other comorbidities such as diabetes, kidney disease, hematological disorders, liver, lung, and neurological pathology (2,3).HG manifests itself from the 20th week of gestation onwards and increases the risk of PE and eclampsia. The latter put the life of the fetus and the mother at risk as they are correlated with stroke and pulmonary edema (PE), which are the significant causes of mortality in this pathology. The HG approach involves changes in lifestyle and eating habits in addition to medication use. At the however, the use of medicines during the gestational period is complex since many of the drugs available go beyond the placental barrier and reach the bloodstream of the embryo, which, as it does not have a fully developed body system, is subject to adverse effects (6). Given this scenario, it is necessary to search for therapeutic alternatives for the treatment of DHG. In recent years, the increased demand for integrative and complementary practices (PIC) in the prevention and treatment of various diseases led the Organization World Health Organization (WHO) asks member states to formulate laws to integrate these practices into their health systems. THE Osteopathy is part of the list of PICs recognized in several countries, including Brazil. Osteopathic manual treatment (BMT) is configured as a non-invasive therapy without the use of drugs, where the professional diagnoses and treats somatic dysfunctions through the hands (6). The disorders treated by osteopathy are cataloged as diseases of the musculoskeletal system (ICD-10, M99.00-09) and identified by the TART parameters (Tenderness, Asymmetry, Range of motion change, Tissue texture change) (7). The importance of increasing research about OMT in pregnant women is due to the need to find safe and effective treatment practices during pregnancy, especially in high-risk pregnancies (8–10).

**Hypothesis:**

Osteopathic manipulative treatment alters the hemodynamics of pregnant women

**Primary Purpose:**

To evaluate the hemodynamic effects of OMT on the mean BP of pregnant women.

**Secondary Purpose:**

To compare the effects of OMT on maternal-fetal vascularization between groups of pregnant women.

Changes in quality of life of pregnant women through the SF-36 quality of life questionnaire.

Perinatal outcomes among the group of pregnant women.

**Proposed Methodology:**

This is a cohort study with a convenience sample of normotensive and hypertensive pregnant women followed at the prenatal clinic and in the IFF/Fiocruz pregnant women's ward. The prenatal service at the IFF “takes care of pregnant women with a gestational age of less than 24 weeks, without clinical comorbidities and with indications such as infectious diseases with risk of fetal repercussion, adolescents aged Fifteen years, history of previous malformation pregnancies, maternal age 35 years or older, multiple gestations, hemolytic disease perinatal, and maternal gynecological malformation. "The services will take place at the Fernandes Figueira Institute, a unit of the Oswaldo Cruz Foundation dedicated to teaching, research, and technological development. The Institute is recognized as a reference center for the health of women, children, and adolescents. It has the “MS assignment of assisting, coordinating and evaluating actions aimed at women's and children's health within the scope of national." The research will be composed of two groups: a control group formed by normotensive pregnant women and an exposed group of pregnant women hypertensive. The appointments will take place on Tuesdays and Thursdays at the prenatal outpatient clinic or the pregnant women's ward of the IFF, with all pregnant women who agree to participate in the research. These will be met with OMT from their entry into the study until the time of termination of pregnancy. OMT consultations will occur at each routine visit of these pregnant women to the outpatient clinic. Pregnant women will respond to a standard form prepared by the researchers in addition to a life questionnaire, the SF-36, applied after each meeting. The average duration of consultations takes around 30 to 40 minutes, and the techniques chosen are widely recognized by osteopathic professionals and described in articles as safe practices. There will be no maneuvers on the patient's abdomen, just as there will be no maneuvers on the viscera, as it is not a practice of visceral osteopathy. Before and after each visit, the BP of the pregnant woman will be evaluated using a blood pressure device. OMRON HEM-7320F, validated for research by the Brazilian College of Cardiology1F. BP will always be measured on the left arm, with the patient sitting and in left lateral decubitus. Pregnant women who are hospitalized in the ward, they will also be treated with the same OMT but will have a different measurement. Before the OMT observes, the data of blood pressure (BP), heart rate (HR), and respiratory rate (RR) are present in the electronic monitor (without any interference by the human operator). An ultrasound (US) will be performed for fetal evaluation through the doppler of the uterine, umbilical and cerebral arteries, and then the same life questionnaire (QoL) will be applied. Only after these measurements will OMT occur. After 30 minutes of the intervention, the blood pressure, heart rate, and respiratory rate data on the electronic monitor will be measured again (without interference from the human operator). A new ultrasound will be performed for fetal evaluation through the doppler of the uterine, umbilical and cerebral arteries. One recent evaluation will be carried out 24 hours after OMT. Blood pressure, heart rate, and respiratory data will again be observed on the electronic display (without any interference by the human operator). Another ultrasound will be performed for fetal evaluation through the doppler of the uterine, umbilical and cerebral arteries, and then the same life questionnaire will be applied. The OMT protocol includes techniques for balance ligament (BLT), myofascial, muscular energy, and cranial tensions. High-velocity and low-speed (HVBA) styles were excluded. Pregnant women in both groups will maintain standard obstetric and drug treatment and must sign a free-term consent, their willingness, and awareness of participating in the research.

**Inclusion Criteria:**

**Inclusion criteria:** the sample from normotensive and hypertensive pregnant women in the third trimester, over 18 years old, and accepted at the IFF/Fiocruz to carry out prenatal care at the outpatient clinic and in the pregnant women's ward from July 2021 to September 2022. The minimum age of patients was defined according to the physiological differences observed between adolescent and adult pregnant women.

**Exclusion Criteria:**

**Exclusion criteria:** pregnant women with bleeding, with clear signs of premature birth (contraction, dilation), with pregnancy

twin, with fetal malformation, signs of eclampsia (based on blood tests, urine, and BP measurement), and HELLP syndrome.

**Risks:**

The risks of osteopathic treatment are small, especially with what we will do with pregnant women who are willing to participate in the search. The possible risk of feeling a little limp or a little dizzy. If she feels something different during the treatment, the pregnant woman must inform that we can decide whether or not to stop the treatment. Stopping therapy is usually enough to clear up any discomfort.

**Benefits:**

Osteopathic manipulative treatment (OMT) has been proven to help with painful processes in pregnant women. We are looking for a search aid in BP control in hypertensive pregnant women, which will be possible if the response to treatment is positive.

**Data Analysis Methodology:**

Relative risk (RR) and differences in means will be the measures of association used to assess the relationship between BMT and changes in hemodynamics as well as fetal outcomes. Categorical variables will be evaluated using the chi-frame test and numeric variables using the t-student test. The data will be digitized in the database with the help of Epi INFO and analyzed in SPSF

**Primary Outcome:**

The evolution of hemodynamic parameters during the third trimester of pregnancy

**Secondary Outcome:**

Mean birth weight and gestational age at birth and the frequencies of resuscitation, NICU admission, and death

fetal or within the first 12 hours. These results will be controlled by the beginning of prenatal follow-up, maternal age, and weight gain.

gestational.

**Sample Size in Brazil:** 80

**Recruitment Countries**

**Country of Origin of the Study Number of research participants**

**Recruitment Countries**

**Country**

BRAZIL

**Other information**

be using secondary data sources (medical records, demographics, etc.)?

yes

**Details:**

Clinical and demographic data of the pregnant women will be observed (age, education, professional situation, ethnicity, smoking, alcoholism, number of children, number of pregnancies, number of abortions, number of births, the practice of physical activities, BMI at the beginning of pregnancy); obstetrics (date of last menstruation, diabetes, kidney pathologies, chronic respiratory pathologies, musculoskeletal pain, cardiac pathologies, diseases autoimmune disorders, a medication used, infection, gestational weight gain, abdominal pain, bleeding); maternal hemodynamics (blood pressure diastolic, systolic blood pressure, and heart rate); and perinatal (ruptured pouch, membrane rupture, chorioamnionitis, meconium, type of delivery, fetal death, birth weight, gestational age, weight-for-age adequacy, head circumference, length, weight z-score, weight z-score length, HC z-score, 1st, and 5th-minute Apgar, resuscitation, admission to the neonatal ICU, and death within the first 12 hours).will be observed from the medical records at each consultation of the patients in the prenatal clinic and recorded in a specific clinical history of the study for subsequent registration in the database.
