# Supplementary material for: Effects of osteopathic manipulative treatment on maternal-fetal hemodynamics in third trimester pregnant women: A prospective study
Source: PLoS One. 2024 Mar 20;19(3):e0300514. doi: 10.1371/journal.pone.0300514 (PMC10954147; doi:10.1371/journal.pone.0300514)
Supplement: S2 File — (DOCX) [file pone.0300514.s004.docx]

## **S2-Support Information - OMT protocol:**

The protocol prepared for the study, included osteopathic techniques for balancing ligament tensions (BLT), myofascial, muscle, and cranial energy. High-velocity, low-amplitude (HVLA) techniques were excluded. The OMT sessions ranged from 30 to 40 minutes and the pregnant women were instructed to maintain standard obstetric and pharmacological treatment.

**1- Upper thoracic opening- BLT –** The patient is seated with the practitioner behind and hands leaning on the patient’s scapular girdle. The practitioner positions the thumbs on either side of the C7 spinous process and the fifth fingers to spread the shoulder from the acromioclavicular joint, with the other fingers on the clavicles (1).

**2- Stretching of the pleural dome ligaments –** Patient seated on the edge of the bed, with the practitioner standing behind, arm on the side to be treated on the practitioner’s leg, in 30⁰ abduction and head in inclination/rotation opposed to the arm in abduction (2).

**3**- **Relaxation and stretching of the lateral lumbocostal arch –** Patient and practitioner sitting side by side. The thumb of the practitioner’s caudal hand is situated below the patient’s 12th rib. Initially, the patient’s trunk is moved to the opposite position, then homolateral inclination when the thumb is pressed in the body’s medial direction (2).

**4- Thoracic diaphragm technique–**patient in lateral decubitus, the practitioner is behind the patient’s lower torso. The abdominal hand gently settles into the softer part of the belly, above the pregnant uterus. The abdominal hand uses the side of the index finger as the guiding contact rather than the fingertips. Once the abdominal hand has settled slightly under the costal margin, it rests there a moment whilst the practitioner engages the lower ribcage with the other hand. With the hands now slightly overlapped, this relationship is maintained throughout the technique’s rest and release of the tension (3).

**5- The technique for lumbar spinal muscles-** The patient is in the lateral decubitus position, and the practitioner faces the patient. Place the two hands superimposed on each other in contact with the lumbar musculature. It will pull the muscle mass in all directions to release tension. Do it bilaterally (4).

**6- BLT lumbosacral junction, pelvis, and hip -** The patient is seated with feet held by the practitioner and knees extended. the patient should not bend backward. The practitioner then moves the legs towards ease, and the legs are held in that position. While the legs are being held in this position, the patient is asked to slowly rotate the trunk to the facilitated side until the sacrum begins to move. At that moment, the patient stops turning and remains in this position until the tensions are relaxed (1).

**7- Elevation of the pelvic floor in lateral decubitus –** the seated practitioner places the elongated fingers on the patient’s pelvic floor, between the ischiatic tuberosity and the rectum. Gently enter their fingertips in the cephalic direction during the expiration phase (2).

**8- Decompression of the atlantooccipital joint –** the practitioner makes contact close to the occipital condyles, leading to anterior support, added to lateral and cephalic tension (5).

**9- Treatment of the floor of the mouth -** the practitioner places their fingertips in a medial position to the jawline and applies uniform pressure on both sides to balance the existing muscular tensions. Stop when your fingers perceive that the tissue has softened (6).

**10- Alternating rotation of the temporal –** Gentle compression on the mastoids to move them in the opposite direction (5).

**References:**

1. Carreiro J- *Paediatric Manual Medicine, an osteopathic approach,* Churchill Livingstone, Elsevier, 2012, UK.
2. Coster M, Pollaris A- *Osteopatía Visceral,* Editorial Paidotribo, 2001, Barcelona.
3. Stone, Caroline A- *Visceral and Obstetrics Osteopathy*- Churchill Livingstone/Elsevier- 2007.
4. Sandler S- *Osteopathy and obstetrics,* Ansham Ltd, reprinted 2016.
5. Magoun, Harold I**-** *Osteopathy in the cranial field*- third edition, Sutherland Cranial Teaching Foudation,1976.
6. Bordoni, B e Zanier, Emiliano- *The continuity of the body: hypothesis of treatment of the five diaphragms*- The Journal of Alternative and Complementary Medicine-2015.
